# Retraction: The Bicarbonate Transporter Is Essential for *Bacillus anthracis* Lethality

**DOI:** 10.1371/annotation/26144e46-0873-48d9-890f-7ae3136a79d4

**Published:** 2010-03-16

**Authors:** Adam C. Wilson, Magali Soyer, James A. Hoch, Marta Perego

Upon re-examining the results presented in this paper and following the rebuilding of the strains described, we were unable to reproduce the dependence of toxin production on the transporter described in the paper. This is likely because of a secondary mutation present in the original strain. Therefore, the conclusions reached in the paper should not be taken as fact, and the paper must be retracted. The decision to retract this paper was reached in consultation with the journal's editors, and the purpose of the retraction is to correct the literature. We hope to uncover the exact reasons for these discrepancies by finding the source of the secondary mutation.

